# Supplementary material for: Power Contours: Optimising Sample Size and Precision in Experimental Psychology and Human Neuroscience
Source: Psychol Methods. 2020 Jul 16;26(3):295–314. doi: 10.1037/met0000337 (PMC8329985; doi:10.1037/met0000337)

Open Practices Disclosure

Manuscript Title:
Author Names (in order of authorship):
Corresponding Author:

Articles are eligible to earn badges that recognize open scientific practices: publicly available data, material, or preregistered research plans. You can read more about the badges on the Open Science Framework wiki.

To apply for one or more badges acknowledging open practices, please check the box(es) corresponding to the desired badge(s) below and provide the information requested in the relevant sections. To qualify for a badge, you **must** provide a URL, doi, or other permanent path for accessing the specified information in a public, open-access repository, and the specified information must be provided in a format that is time-stamped, immutable, and permanent. **Qualifying public, open-access repositories are committed to preserving data, materials, and/or registered analysis plans and keeping them publicly accessible via the web in perpetuity.** Examples include the Open Science Framework (OSF) and the various Dataverse networks. Hundreds of other qualifying data/materials repositories are listed at http://re3data.org/. Preregistration of an analysis plan must take place via a publicly accessible registry system (e.g., OSF, ClinicalTrials.gov or other trial registries in the WHO Registry Network, institutional registration systems). **Personal websites and most departmental websites do not qualify as repositories.**

Authors who wish to publicly post third-party material in their data, materials, or preregistration plan must have the proper authority or permission agreement in order to do so.

There are circumstances in which it is not possible or advisable to share any or all data, materials, or a research plan publicly. For example, there are cases in which sharing participants' data could violate confidentiality. If you would like your article to include an explanation of such circumstances and/or provide links to any data or materials you have made available—even if not under conditions eligible to earn a badge—you may write an alternative note that will be published in a note in the article**.** Please check this box if you would like your article to include an alternative note and provide the text of the note below:

[] Alternative note:

The title and author fields above are not possible to fill on my computer. The details are:

Title: Power contours: optimising sample size and precision in experimental psychology and human neuroscience

Authors: Daniel H Baker, Greta Vilidaite, Freya A Lygo, Anika K Smith, Tessa R Flack, Andre D Gouws & Timothy J Andrews

Corresponding author: Daniel H Baker, daniel.baker@york.ac.uk

■ Open Data Badge

Provide the URL, doi, or other **permanent path** for accessing the time-stamped and immutable data in a **public, open-access repository**:

10.17605/OSF.IO/EBHNK

■ Confirm that there is sufficient information for an independent researcher to reproduce **all of the reported results**, including codebook if relevant.

Yes

■ Open Materials Badge

Provide the URL, doi, or other **permanent path** for accessing the time-stamped and immutable materials in a **public, open-access repository**:

10.17605/OSF.IO/EBHNK

■ Confirm that there is sufficient information for an independent researcher to reproduce **all of the reported methodology**.

Yes

☐ Preregistered Badge

1. Provide the URL, doi, or other **permanent path** to the time-stamped and immutable registration in a **public, open-access repository*:**

2. Was the analysis plan registered prior to examination of the data or observing the outcomes? If no, explain.**

3. Were there additional registrations for the study other than the one reported? If yes, provide links and explain.*

4. Were there any changes to the preregistered analysis plan for the primary confirmatory analysis? If yes, explain**

5. Are all of the analyses described in the registered plan reported in the article? If no, explain.*

*No badge will be awarded if (1) is not provided, **or** if (3) is answered "yes" without strong justification, **or** if (5) is answered "no" without strong justification.

**If the answer to (2) is "no," the notation DE (Data Exist) will be added to the badge, indicating that registration postdates realization of the outcomes but predates analysis. If the answer to (4) is "yes" with strong justification for changes, the notation TC (Transparent Changes) will be added to the badge, indicating that the analysis plan was altered but the preregistered analyses and rationale for the change are provided.

By signing below, authors affirm that the above information is accurate and complete, that any third-party material has been reproduced or otherwise made available only with the permission of the original author or copyright holder, and that publicly posted data do not contain information that would allow individuals to be identified without consent.

Signature:

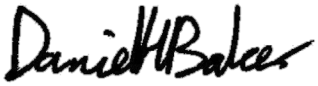

Supplement: Supplementary file 1 [file met0000337_open-practices-disclosure-form.pdf]
